# Withstand Context: Standing Posture Improves Contextual Cueing in Challenging Visual Search

**DOI:** 10.1111/psyp.70108

**Published:** 2025-07-17

**Authors:** Artyom Zinchenko, Nuno Busch, Gordon Dodwell, Thomas Geyer

**Affiliations:** ^1^ Department of Psychology Ludwig‐Maximilians‐Universität München Munich Germany; ^2^ School of Management Technische Universität München Munich Germany; ^3^ School of Psychological Sciences, Birkbeck University of London London UK; ^4^ Munich Center for NeuroSciences—Brain & Mind Planegg‐Martinsried Germany; ^5^ NICUM—NeuroImaging Core Unit Munich Munich Germany

**Keywords:** body posture, contextual cueing, divided attention, executive functions, HRV, load theory, selective attention, sit‐stand, statistical learning, working memory

## Abstract

Humans can learn to use repeated spatial arrangements of irrelevant, non‐target items to direct the focus of attention towards behaviorally relevant—target—items, a phenomenon known as contextual cueing (CC). However, whether CC is itself dependent on attentional resources is a controversial issue. Here, we used visual search to test how CC is affected when attention varies through two types of manipulations: *perceptual load* (as induced by target‐distractor similarity) and *postural load* (sitting vs. standing). For easy searches (low target‐distractor similarity), we observed reliable facilitation of search in repeated‐context displays, which was independent of participants' body posture. For difficult searches (high target‐distractor similarity), contextual facilitation was evident only with standing posture. Posture‐related benefits remained significant even after controlling for heart rate variability (HRV), body mass index, and physical activity. Decomposing aggregated reaction times by drift‐diffusion modeling revealed that CC in difficult searches decreased the amount of evidence required for target‐response decisions. Our results suggest that statistical learning is effectively supplemented during standing posture when visual search is challenging, possibly because posture manipulation and contextual manipulation affect common response‐selection stages of processing.

## Abstract (de)

Standing posture restores and even enhances the well‐known benefits of contextual cueing—where repeated arrangements speed visual search—under challenging conditions. This study advances our understanding of the interplay between bodily states and cognition by revealing how upright posture lowers decision thresholds and improves response selection when target‐distractor similarity is high. These findings highlight the potential for simple postural changes to bolster attentional and memory processes in difficult visuospatial tasks.

## 1 Introduction

We often navigate through complex visual environments without much effort, relying on past experiences to guide our behaviors. For instance, when walking into a familiar grocery store, we almost reflexively know where to find our favorite items, even though we're not actively thinking about their locations. This phenomenon, where repeated exposure to consistent spatial contexts enhances our ability to process behaviorally relevant target items, is known as contextual cueing (CC; e.g., Chun and Jiang 1998). CC demonstrates that visual search is facilitated; that is, search reaction times (RTs) are expedited when the searched‐for target item is consistently located within a stable configuration, or *context*, of non‐target or distractor elements (see, e.g., Zinchenko et al. 2020; Chen et al. 2024; Vadillo et al. 2021). There are two (main) accounts of the mechanism of how RT benefits emerge from experience with repeated displays. One assumes that display repetitions facilitate *response‐selection* stages of processing when participants decide on the orientation of a located target and, consequently, on which motor effector (hand) is required for a correct response (e.g., Kunar et al. 2007; Hout and Goldinger 2010; Schankin and Schubö 2010). The other, *attentional* account, assumes that CC arises because the acquired target‐distractor spatial associations (stored in long‐term memory, LTM) come to guide search, predicting or “cueing attention” to the target location—facilitating the process of locating the target before response selection can take place (e.g., Chun and Jiang 1998; Geyer et al. 2010; for reviews, see, e.g., Goujon et al. 2015; Sisk et al. 2019).

### 1.1 CC, Mental Load, and Working Memory

Chun and Jiang (1998) proposed that CC reflects incidental, that is, automatic, learning. However, later research has shown that manipulations of attention or mental load can influence the CC effect. For example, Annac et al. (2019) demonstrated that when participants were required to judge the quadrant in which the target appeared—using a generation task in which the target in the repeated display is later replaced by a distractor (see, e.g., Chun and Jiang 2003; Geyer et al. 2020)—their ability to detect the target quadrant improved if they could direct focal attention to that specific area. Additional studies have revealed that CC is not limited to stimuli that are initially task‐relevant. In fact, stimuli that are irrelevant during the initial learning phase can produce a CC effect when they become task‐relevant (and thus attended) during a subsequent test phase (e.g., Jiang and Chun 2001; Jiang and Leung 2005; Zang et al. 2022).

**FIGURE 1:**
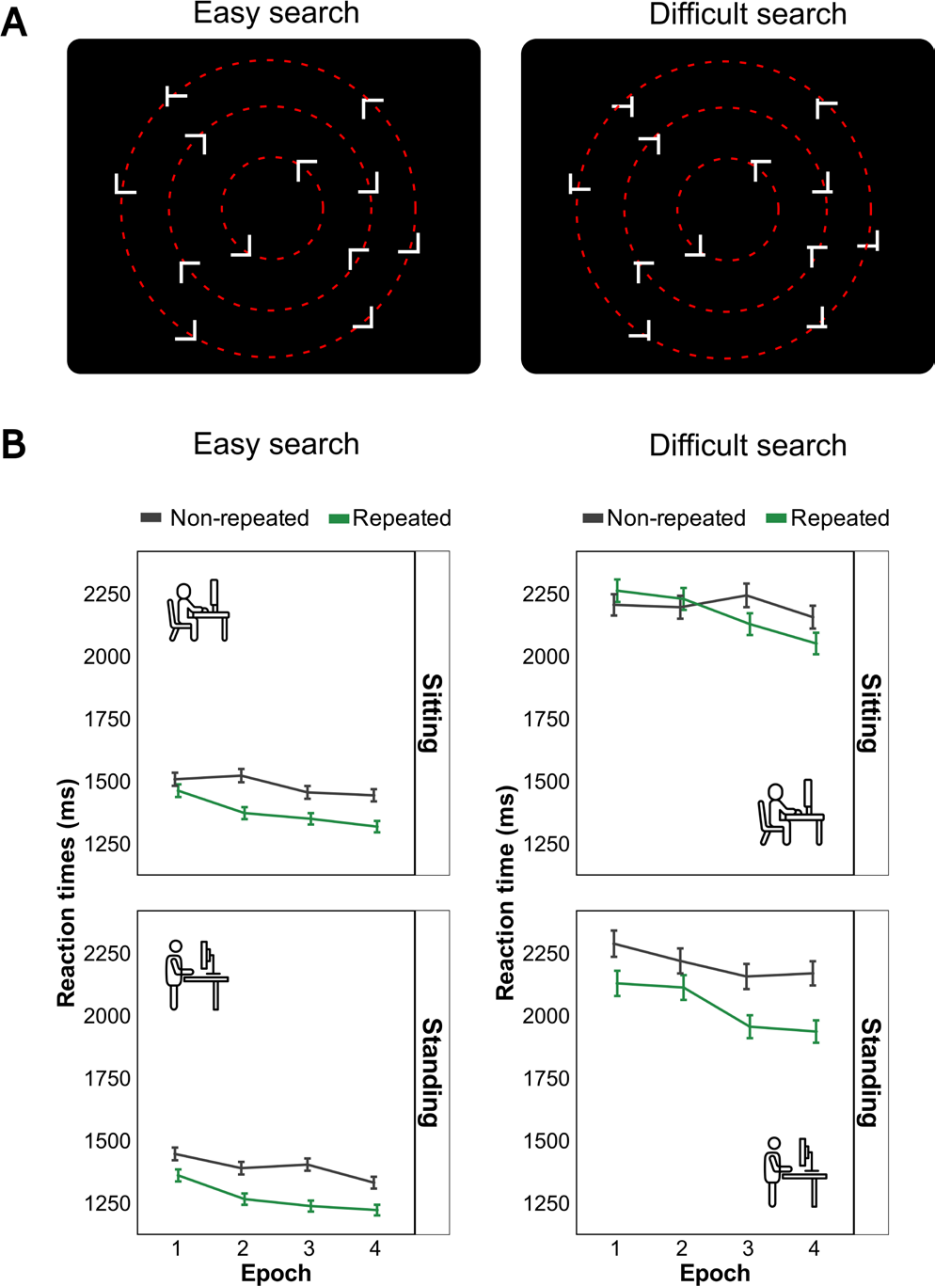
(A) Example images in the easy‐search and difficult‐search tasks. The red dashed circles highlight the placement of the search items, though these circles were invisible in the actual experiments. (B) Mean reaction times (in milliseconds, ms) in repeated and non‐repeated search arrays when target‐distractor similarity is relatively low (=easy‐search Experiment 1a) or relatively high (= difficult‐search Experiment 1b) and when participants performed the search task in sitting or standing position. Error bars represent standard errors of the mean.

In line with these findings, increasing the perceptual similarity between target and distractor items reduces CC (e.g., Lie 2023; Zinchenko et al. 2022; Darby et al. 2014; but see Vaidya et al. 2007). This effect is thought to arise because greater target–distractor similarity increases the demands on representational precision needed to discriminate targets from non‐targets. As a result, more attentional and perceptual “bandwidth” is allocated to basic discrimination processes (i.e., increased perceptual load), leaving fewer resources available for incidental contextual learning. In these (perceptual load) studies, CC is commonly tested using visual search tasks in which participants identify a T target letter among distractor L letters. Perceptual load is modulated by manipulating the point at which the vertical and horizontal lines in the target letter (L or T) intersect, thereby increasing or decreasing target–distractor similarity (see Figure 1A for illustration; for similar approaches, see, e.g., Jiang and Chun 2001; Zang et al. 2015). A common finding is that CC is weaker with high relative to low target–distractor similarity (e.g., Lie 2023).

To understand this phenomenon, we can turn to theories about how visual search operates: According to one account, visual search is guided by working memory (WM) representations (i.e., attentional templates, e.g., Ort and Olivers 2020) that are activated prior to search and contain target‐defining features (e.g., orientation, shape, etc.; cf. Wolfe and Horowitz 2017). Research has demonstrated that the utility of search‐guiding templates depends on target‐distractor similarity, such that participants represent a target item that is difficult to distinguish from non‐target items with greater precision (e.g., Schmidt and Zelinsky 2017; Rajsic et al. 2017), though this comes at the cost of increased WM demands (e.g., Machizawa et al. 2012; Merkel et al. 2021; Luria and Vogel 2011). This WM load becomes particularly relevant in CC because retrieved information from long‐term (LT) memory must first be brought back into WM to become available in the visual search display. Specifically, it has been suggested that WM acts as the “arena” that links information processing between contextual LT memory and visual search (e.g., Annac et al. 2018; Woodman et al. 2013; Conci et al. 2023). In fact, research has shown that when a demanding secondary WM task is introduced during a visual search task with repeated (vs. non‐repeated) displays, the CC effect tends to vanish and only recovers immediately once the WM task is removed (e.g., Annac et al. 2013; Manginelli et al. 2013; see also Vickery et al. 2010). This demonstrates a potential causal role of WM as a linking mechanism between LT memory contextual representations and their use in visual search.

### 1.2 Attention and Body Posture

Assuming a critical role of WM in CC, it becomes important to consider factors that might influence WM performance and the central executive system. One such factor, which has received a great deal of interest in recent years, is an individual's physical state, such as body posture. The available evidence suggests that body posture is an important factor in executive functioning, including WM (e.g., Busch et al. 2025; Abou Khalil et al. 2023, 2020). Evidence from two established experimental paradigms demonstrates the effects of posture on cognitive performance. First, it has been demonstrated that in the Stroop task (e.g., Stroop 1935), participants show faster RTs to incongruent stimuli when performing the color‐naming task while standing versus sitting (e.g., Rosenbaum et al. 2017; but see Busch et al. 2025; Straub et al. 2022). Second, in visual search tasks (e.g., Wolfe 1998), participants locate targets more quickly when standing upright versus sitting down (e.g., Smith et al. 2019; Janssen et al. 2014; but see Caron et al. 2023).

Building on the established relationship between executive functions and WM (e.g., Baddeley 2010), we propose that postural states such as standing (vs. sitting) may enhance executive WM functions (Dodwell et al. 2019; Abou Khalil et al. 2020)—including also (more) effective retrieval from contextual LTM. As a result, two things can happen: Firstly, according to the attention‐guidance account of CC (e.g., Zinchenko et al. 2020), contextual information held in WM could help in guiding attention quickly to the likely target location in the repeated array. Secondly, according to the response‐facilitation account (e.g., Kunar et al. 2007), effective maintenance of contextual representations in WM could facilitate manual responses to the target after it has been detected in the repeated search array. These two hypothesized effects lead to our central question: How does standing influence CC in perceptually easy and difficult search tasks?

### 1.3 Rationale of the Present Study

To test this, in the current study, we recorded behavioral RTs to examine visual search in repeated versus non‐repeated (T vs. L's) letter arrays with high compared to low perceptual load by using manipulations of the offset of the vertical and horizontal lines in the letter L distractors (Figure 1A; between‐subjects factor). Second, we incorporated a posture manipulation (standing vs. sitting) as a within‐subject factor to examine how standing‐induced activations impact CC across the two task difficulty levels. Two groups of 20 participants completed either an “easy” or “difficult” search task with standing and sitting positions across two sessions (minimum 2‐day separation).

Moreover, we applied drift‐diffusion modeling (DDM; e.g., Ratcliff 1978) to separate different sources of variability to account for differences in (aggregated) RTs in our experimental design. The diffusion model assumes that evidence for, for example, a left‐ versus right‐oriented target response accumulates over time with (1) a mean accumulation drift rate, *v*, until (2) one of two decision thresholds is reached (corresponding to the choice options), that are separated in distance as indexed by the boundary separation parameter *a*.

The drift‐rate parameter *v* encapsulates the quality of the evidence driving decision‐making: higher drift rates correspond to stronger, more discriminative evidence entering the decision process. It is important to note that the present study (and prior CC studies) mainly used a difficult T versus L letter search task that requires serial attention for distinguishing a target from non‐target elements (e.g., Wolfe 2021). In this experimental setup, where choice and RT data stem solely from the target orientation decision (left vs. right), distinguishing whether drift rate effects arise from enhanced target identification among distractors or improved judgment of the target's orientation after identification is challenging. Consequently, we attribute the drift‐rate parameter to the perceptual accumulation of “target” vidence, that is, including both evidence regarding the various items' status as target versus nontarget (pre‐attentive target selection) and evidence as to the target's response‐critical feature (post‐selective target identification, e.g., determining the visual target as left‐ or right‐oriented). Accordingly, one prediction coming particularly from the attention‐guidance account of CC (e.g., Chun and Jiang 1998) is that the drift‐rate parameter is increased in old‐context (vs. new‐context) displays. However, the literature varies considerably in how the drift rate is mapped to theoretical CC process stages—whether it reflects target location or response selection (e.g., Sewell et al. 2018; Chen et al. 2021). We revisit this issue in the discussion.

The boundary separation *a* represents the distance between the two decision thresholds, indicating the amount of evidence needed to decide on a response (e.g., Ratcliff et al. 2015). Observing a reduced boundary separation would mean that less evidence must be accumulated to reach a decision (i.e., the decision criterion is set more liberally), thus expediting the selection of responses (even if the drift rate was constant). Accordingly, one prediction for this parameter, particularly from the response‐threshold account of CC (e.g., Kunar et al. 2007), is that the boundary separation is lowered in old‐context (vs. new‐context) displays.

Notably, previous studies that applied the drift‐diffusion model to a visual search task with an old/new context manipulation found that in the standard T versus L's paradigm (with low target‐distractor similarity), learned contextual cues improve both the drift rate and boundary separation parameter (e.g., Sewell et al. 2018; Weigard and Huang‐Pollock 2014). We can thus assume that these parameters aptly indicate the (more effective) search‐ and response‐related processes that can lead to the CC effect.

Our particular focus was on quantitatively investigating participants' drift‐diffusion model parameters (and RTs) in old‐ and new‐context displays when visual letter search is easy or difficult and performed in standing versus sitting conditions. This could reveal new information about whether diminished CC with high load compared to low load search (e.g., Lie 2023; Darby et al. 2014) is caused by changes in the drift rate parameter or boundary separation parameter (or both). Moreover, we could investigate an even more important question, namely, whether a posture manipulation that is assumed to promote executive processing (e.g., Rosenbaum et al. 2017; Busch et al. 2025; Dodwell et al. 2021) exerts an effect on CC and if so, whether this is due to improvements in the drift rate and/or boundary separation parameter/s. We also measured aerobic fitness indices by means of body mass index (BMI), physical activity (PA) self‐reports, and cardiac arousal measures. These (cardiac arousal) measures were obtained to rule out arousal‐based explanations for the observed contextual‐cueing effects (e.g., Busch et al. 2025; Abou Khalil et al. 2023). As our main hypothesis focused on how postural state affects CC, it was critical to rule out the possibility that any observed differences in behavioral or model‐based measures were simply due to increased *physiological* arousal in the standing condition. Therefore, cardiac arousal measures were included as covariates in our mixed‐effects behavioral models but were not intended to index trial‐wise or participant‐wise fluctuations. The study was not preregistered.

## 2 Methods

### 2.1 Participants

A total of 41 participants took part in the study. One person could not attend the second session, leaving a final sample of *N* = 40 participants (i.e., *N* = 20 participants in each Experiment). Experiment 1a (easy search) included 14 female and 6 male participants (*M* = 23.95 years, SD = 3.25). Experiment 1b (difficult search) included 9 female and 11 male participants (*M* = 27.20 years, SD = 6.10).^1^ A power calculation using the R “pwr” package (Champely et al. 2017) showed that *N* = 40 participants are sufficient to observe a *context × task difficulty* interaction reported, for example, in Darby et al. (2014) with > 91% power (see also Geyer et al. 2024). Participants were not eligible if they reported mental health conditions, used prescription medications that could affect cognitive or motor function, had difficulties maintaining seated or standing positions, or had any neurological disorders. All participants had either normal or corrected‐to‐normal vision. The LMU's Psychology Department ethics committee gave approval for the current study, which was supported by a grant from the German Research Council (DFG; see Funding information). Before participating, each participant provided written informed consent. The study procedures followed the ethical principles outlined in the Declaration of Helsinki.

### 2.2 Apparatus and Stimuli

The experimental routine was programmed in Matlab with Psychtoolbox extensions (Brainard 1997; Pelli and Vision 1997) and was run on a PC under the Windows 7 operating system. Participants were seated in a dimly lit room in front of a 23‐in. LCD monitor (ASUS, Taiwan; refresh rate 60 Hz; display resolution: 1920 × 1080 pixels) at a viewing distance of approximately 80 cm (unrestrained). The search displays consisted of 12 dark‐gray items (luminance: 1.0 cd/m^2^): 1 T‐shaped target and 11 L‐shaped distractors presented against a black background (0.11 cd/m^2^). The offset at the junction of the L distractor shapes was 0% of the letter height in the easy condition and 11% in the difficult condition. All stimuli extended 0.35° of visual angle in width and height. The items were arranged on three (invisible) concentric circles around the display center (with radii of 1.74°, 3.48°, and 5.22° for circles 1 through 3, respectively). The target was always positioned on the second circle (see Figure 1).

There were 16 possible target locations, eight of which were used for repeated displays with constant distractor layouts, and another set of eight target locations was used for non‐repeated displays with randomly composed distractor layouts. For each set of target locations per context condition (repeated and non‐repeated displays), two targets were presented in each of the four display quadrants. The “T” target was rotated randomly by 90° to either the left or right. The 11 remaining items were L‐shaped distractors rotated randomly at orthogonal orientations (0°, 90°, 180°, or 270°). Importantly, participants were not informed that some search arrays were presented repeatedly.

### 2.3 Trial Sequence

A trial started with the presentation of a fixation cross (size: 0.10°, luminance: 1.0 cd/m^2^) for 500 ms, followed by a blank screen interval of 200 ms before the onset of the search display. Participants were instructed to respond as quickly and accurately as possible to the orientation of the “head” of the T (left vs. right). Each search display stayed on the screen until the observer's manual choice response was registered. Participants responded by pressing the left/right arrow button on a computer keyboard with their corresponding index finger. Following an erroneous response, the word “Wrong” appeared on the screen for 1500 ms. The intertrial interval was set at 1000 ms following a correct response. Each experiment consisted of 25 blocks × 16 trials per block, yielding 400 trials in total. Participants could take a short break between blocks or continue directly with the next block. To acquire reliable estimates of CC, defined as the difference in RTs between repeated and non‐repeated displays, we assigned each trial to an epoch corresponding to five consecutive blocks (see, e.g., Chun and Jiang 1998, for a similar approach).

### 2.4 Task Procedure

The repeated context condition comprised eight randomly arranged target‐distractor configurations generated at the beginning of the respective experiment. These arrangements were repeatedly presented on randomly selected trials throughout each block of the search task, with the restriction that each repeated display was shown only once per block. Displays in the non‐repeated condition were generated at the beginning of a given trial. Repeated arrangements were presented in half of the trials, and non‐repeated arrangements in the other half. The trial order was randomized in each block. To equate target location repetition effects between the two types of displays, the target appeared equally often at each of 16 possible locations throughout the experiment: eight locations were used for repeated displays, and the remaining eight for non‐repeated displays. Please note that for repeated displays, the spatial configuration of targets and distractors was held constant across all trials, though the orientation of the target varied randomly from trial to trial.

Participants performed a visual search in repeated and non‐repeated displays (within‐subject factor) with either high or low target‐distractor similarity (between‐subject factor) and in either standing or sitting posture (within‐subject factor). The posture conditions were counterbalanced across the participants to control for order effects. The mean interval between sessions was 2.59 days (SD = 1.22 days). We also included several aerobic control variables: during their first session, participants provided demographic data and anthropometric measurements for BMI calculations in addition to completing the short version of the International Physical Activity Questionnaire (IPAQ; Craig et al. 2003).

### 2.5 Recognition Test

At the end of the experiment, participants performed a yes/no recognition test to examine whether they had explicit memory of the repeated configurations (e.g., Chun and Jiang 1998). Eight repeated displays from the search task and eight newly composed displays were shown, and observers indicated whether they had seen a given display previously. The eight repeated and eight non‐repeated displays were presented in random order. The recognition responses were non‐speeded, and no error feedback was provided.

### 2.6 Heart Rate Measurement

We measured heart rates during sitting and standing conditions using a Polar H10 chest strap sensor (Polar Electro Oy, Finland) connected to an iPhone running HRV+ app (v2.9.2, ZUZ LLC). Data analysis was performed in R using the RHRV package (v4.2.7; Martínez et al. 2017). After removing outliers from the non‐interpolated heart rate (NIHR) data using FilterNIHR(), we interpolated the data at 4 Hz for spectral analysis. We calculated mean NIHR for beats per minute (BPM), SDNN (standard deviation of RR intervals) for heart rate variability (HRV), and the low‐frequency to high‐frequency power ratio (LF/HF; 0.04–0.15 Hz/0.15–0.4 Hz) using 300‐s windows with 30‐s shifts.

These cardiac measures may indicate physiological arousal and autonomic nervous system dynamics. HRV reflects autonomic balance regulation and neurocardiac function (Shaffer and Ginsberg 2017). While affected by factors like posture, resting HRV correlates with executive functioning and adaptability (Bertsch et al. 2012; Thayer et al. 2009). The LF/HF ratio indicates sympathetic‐parasympathetic balance, varying with physiological arousal and postural changes (Perini and Veicsteinas 2003). Due to technical limitations, heart rate measures were recorded independently of the presented stimuli. Since heart activity was measured continuously throughout the entire session of around 45 min, all reported cardiac measures reflect averages computed across this full duration. For three participants, we used an alternative Heart Rate Monitor app (BM Innovations GmbH) running on Android, which did not allow us to compute SDNN or LF/HF ratio and only gave out an average value for BPM.

### 2.7 Assessment of Personal Fitness Characteristics

The short form of the IPAQ asks about individual PA habits in three domains: walking, moderate‐intense activity, and vigorous‐intense activity (Craig et al. 2003). IPAQ data were processed according to the processing and analysis guidelines (IPAQ Research Committee 2005): Weighting each activity type by its energy requirements defined in multiples of resting metabolic rates (METs) to get a MET‐minutes score as a measure of activity volume (equivalent to kilocalories for a 60 kg person). We used the recommended MET weights for each domain (Walking = 3.3; Moderate activity = 4.0; Vigorous activity = 8.0; IPAQ Research Committee 2005) to compute the individual domain‐specific MET minutes/week before summing them up to a total PA MET‐minutes/week score per subject (PA). The BMI was calculated from the self‐reported individual height (l) and weight (m) values using the standard formula $\mathrm{BMI}=\frac{m}{l^{2}}$. One participant's BMI was missing because this person did not want to indicate their weight and height.

### 2.8 Statistical Analysis

To ensure data quality, we excluded RTs exceeding 2.5 standard deviations from each participant's mean and RTs from incorrect responses (see, e.g., Zinchenko et al. 2024). Due to incomplete heart rate measurements for four participants (two in each easy/difficult‐search condition), we employed mixed‐effects models using the R packages lme4 (Bates et al. 2015) and lmerTest (Kuznetsova et al. 2017).

The dependent variable was RT, with fixed effects including the experimental factors of context (“Non‐repeated” and “Repeated”), posture (“Sitting” and “Standing”), difficulty (“easy” and “difficult”), and epoch (1–4). We included continuous covariates of HRV, BMI, and total index of PA. The PA index scores, which exceeded 7000 and were substantially higher than other predictors, were scaled prior to analysis. Other covariates remained unscaled to prevent rank deficiency and convergence issues.

For random effects, we initially specified participant ID with random intercepts and maximal random slopes for context, posture, and difficulty. When more complex structures failed to converge, we adopted a consistent approach across all analyses: random intercepts for each participant with random slopes for posture and difficulty. This strategy ensured both model convergence and analytical consistency.

For direct comparisons of heart rate measures across conditions, we analyzed only complete datasets (*N* = 36) using a mixed‐design ANOVA implemented with the “ez” library in R (Lawrence and Lawrence 2016). Given the generally low error rates in manual responses, which resulted in numerous zero values, we analyzed error rates using zero‐inflated generalized linear mixed‐effects models. These models were implemented through the glmmTMB package in R (Brooks et al. 2017), enabling us to incorporate both physiological measures and fitness parameters as covariates while appropriately accounting for the excess of zeros in our data.

### 2.9 Drift‐Diffusion Modeling

DDM was realized using the Dynamic Models of Choice (DMC) package in R (Heathcote et al. 2019). The model accounted for three key experimental manipulations: difficulty (easy vs. difficult), posture (sitting vs. standing), and context (repeated vs. non‐repeated displays). The diffusion model included the boundary separation (*a*) and drift rate (*v*) parameters that were mapped factorially across the 2 × 2 × 2 experimental conditions, along with, across experimental conditions, constant non‐decision time (*t*_0_), starting point (*z*), starting point variability (*sᶻ*), and drift rate variability (*sᵛ*) parameters. Non‐decision time variability ($s_{t_{0}}$) and differences in response n speed (*d*) were also held constant (at zero) across conditions. Please note that the most appropriate (drift rate × boundary separation) DDM model was identified by a systematic DDM model comparison approach (e.g., Chen et al. 2021; see Section S3).

Model parameters were estimated using Bayesian Markov Chain Monte Carlo (MCMC) sampling. Truncated normal priors were applied for 2 × 2 × 2 boundary separation parameters, 2 × 2 × 2 drift rate parameters, one non‐decision time parameter, and one drift rate variability parameter. Beta distributions were used to determine the starting point and its variability. Parameters were constrained to ensure psychological plausibility. In particular, boundary separation was set to range from 0 to 5, reflecting its role in the speed–accuracy trade‐off, with typical values reported between 0.1 and 2, allowing flexibility for cautious responses (Ratcliff and McKoon 2008; Ratcliff and Tuerlinckx 2002). Drift rates ranged from −5 to 5, accommodating tasks of varying difficulty, as standard values usually range from −2 to +2 (Ratcliff et al. 1999). Drift rate variability was constrained between 0 and 2, reflecting across‐trial differences, with common estimates ranging from 0.1 to 1 (Ratcliff and Rouder 1998). Finally, non‐decision time ranged from 0.15 to 1 s, capturing processes such as stimulus encoding and motor execution (Ratcliff and Tuerlinckx 2002). The beta‐distributed parameters for the starting point (*z*) and its variability (*sᶻ*) naturally fell within [0, 1], as they represent the proportions of boundary separation (Vandekerckhove and Tuerlinckx 2007).

For model fitting, RTs were converted from milliseconds to seconds, and responses were coded as left or right, corresponding to the target orientation. We employed the DMC package's MCMC procedure, initializing each of the 60 chains with 100 burn‐in iterations to facilitate convergence. After this initial phase, we used the run.converge.dmc function, which iteratively added sampling blocks of 10,000 iterations (with a migration probability of 0.05 to mitigate stuck chains) until convergence diagnostics indicated robust mixing. This process resulted in 10,100 total iterations per chain.

We assessed convergence using multiple diagnostics, including the Gelman‐Rubin statistic (multivariate PSRF = 1.01), with individual parameters showing PSRF values of ≤ 1.01. Trace plots (see Figure S1) demonstrated stable mixing across all conditions. We evaluated model fit by comparing posterior predictive distributions to observed cumulative distribution functions of the RTs (see Figure S2). Summary statistics and posterior distribution visualizations further confirmed that the model fits the empirical data well across all experimental conditions.

We employed a Bayesian parameter estimation approach within the DMC framework by computing mean differences between conditions and deriving corresponding 95% credible intervals (CIs). In Bayesian inference, these CIs represent the range of parameter values containing 95% of the posterior probability mass, given the observed data and prior assumptions. If the interval does not include zero, it provides strong evidence that the difference is meaningfully distinct from zero. To further quantify the directional evidence for effects, we calculated Evidence Ratios (ERs)—which can be interpreted as Bayesian directional probability values or Bayesian *p*‐values—after excluding a small Region of Practical Equivalence (ROPE) of ±0.01 (see Choo et al. 2022). This ROPE was chosen to reflect a “practically negligible” difference relative to the typical ranges observed in drift‐diffusion models (approximately 0.03–0.4 for drift rates and 0.08–0.15 for boundary separation; Ratcliff and McKoon 2008; Vandekerckhove et al. 2011).

An ER represents the posterior probability that an effect is in the indicated direction, divided by the probability it is in the opposite direction after accounting for the ROPE. An ER of 1 indicates that the posterior samples (beyond the ROPE) equally favor a positive or a negative effect, whereas values approaching 0 or ∞ indicate stronger directional evidence. For instance, an ER of 0.01 means that 1% of posterior samples (outside the ROPE) favor one direction while 99% favor the other, yielding a 1:99 probability ratio favoring the more probable direction. (Here, the ER is given for the less probable direction; conversely, for the more probable direction, the ER would be 99.) As another example, an ER of 4 indicates that, after excluding the ROPE, 80% of posterior samples support an effect in one direction while 20% support the opposite, corresponding to 4:1 odds. Thus, ERs provide a measure of directional evidence from the posterior distribution, which can be interpreted as Bayesian odds (but not Bayes Factors) for the effect's direction.

Because Bayesian methods do not rely on significance testing in the frequentist sense, we only use terms such as “significant” and “non‐significant” to align with traditional reporting conventions. Specifically, we label an effect “significant” if its 95% CI excludes zero and if its ER strongly favors one direction (i.e., close to 0 or ∞). Finally, please note that while our Bayesian analysis does not require hierarchical testing or corrections for multiple comparisons (as each comparison provides its own complete posterior distribution of evidence), we present our results following the traditional analysis structure of higher‐order interactions followed by simpler effects. This approach aligns with the frequentist analyses used elsewhere in this paper and provides readers with a familiar framework for understanding the pattern of results.

## 3 Results

### 3.1 Physiological Posture Effects

To assess the effects of our postural manipulation, we examined whether physiological arousal (as indexed by the heart rate measures) differed between the 2 (sitting vs. standing) × 2 (easy vs. difficult) conditions of our experimental design. Given that the heart rate measures did not vary across trials (see methods section), a mixed‐design ANOVA was performed with posture (sitting, standing) as a within‐subject factor and search‐task difficulty (easy, difficult) as a between‐subject factor. As can be seen from Table 1, across both difficulty levels, there was a significant *increase* in average BPM and a *reduction* in HRV from sitting to standing posture. Likewise, there was a significant *increase* in the ratio of low‐ versus high‐frequency heart rate measures (LF/HF) from sitting to standing position.

**TABLE 1:** Comparisons of heart rate measures across easy and difficult searches and sitting and standing postures.

Measure	Effect	*F*(1, 34)	*p*	*η* ^2^ᵍ	Condition means
BPM	Difficulty	1.17	0.287	0.027	Sitting‐easy: 85.39
	Posture	34.83	< 0.001*	0.173	Standing‐easy: 96.22
	Difficulty × posture	0.53	0.470	0.003	Sitting‐difficult: 79.38
					Standing‐difficult: 93.28
HRV	Task	0.24	0.628	0.005	Sitting‐easy: 58.58
	Posture	5.39	0.026*	0.040	Standing‐easy: 50.09
	Difficulty × posture	0.04	0.834	< 0.001	Sitting‐difficult: 62.74
					Standing‐difficult: 52.55
LF/HF	Task	0.69	0.411	0.015	Sitting‐easy: 2.89
	Posture	29.42	< 0.001*	0.166	Standing‐easy: 5.59
	Difficulty × posture	0.25	0.618	0.002	Sitting‐difficult: 3.45
					Standing‐difficult: 6.70

Abbreviations: BPM, beats per minute; HF, high‐frequency power; HRV, heart rate variability; LF, low‐frequency power; LF/HF, the ratio of LF and HF.

* *p* < 0.05.

These results demonstrate robust physiological differences between standing and sitting. In particular, the observed cardiovascular adjustments of increased heart rate, decreased HRV, and increased LF/HF ratio are consistent with the expected autonomic responses to orthostatic stress (Srinivasan et al. 2002). Notably, these physiological changes were comparable across both easy and difficult visual search tasks, suggesting that postural effects on cardiovascular measures were reliable and independent of task difficulty.

### 3.2 Psychological Posture Effects I: Error Rates

Given the low overall error rates, we analyzed error data using a generalized linear mixed‐effects model with a zero‐inflated binomial distribution. This approach accounted for the high proportion of zero error trials while allowing us to include context (repeated, non‐repeated), posture (sitting, standing), difficulty (easy, difficult), and epoch (1–4) as fixed effects. Trial‐level data enabled us to capture across‐trial variability and better accommodate the rare and uneven distribution of errors (see Baayen et al. 2008; Barr 2013; Judd et al. 2012). We included HRV, total PA, and BMI as covariates to account for individual differences in autonomic regulation, habitual PA levels, and body composition, respectively. Participant ID was included as a random factor. The analysis revealed a main effect of posture, *χ* ^2^(1) = 6.98, *p* = 0.008, indicating fewer errors in the standing (*M* = 2.47%) compared to the sitting posture (*M* = 2.60%). Further, the posture × difficulty interaction was significant, *χ* ^2^(1) = 5.71, *p* = 0.017. Separate models were run for easy and difficult trials to unpack this interaction. For easy trials, no significant effect of posture was found, *χ* ^2^(1) = 0.16, *p* = 0.687, with similar error rates for the standing (*M* = 1.70%) and the sitting postures (*M* = 1.68%). In contrast, for difficult trials, posture significantly impacted response errors, *χ* ^2^(1) = 13.47, *p* < 0.001, with fewer errors in the standing (*M* = 3.24%) compared to the sitting posture (*M* = 3.52%). This indicates that standing was particularly beneficial in reducing errors during difficult search.

There was also a significant main effect of HRV on error rates, *χ* ^2^(1) = 8.17, *p* = 0.004. Specifically, higher HRV was associated with fewer errors, with an estimate of −0.017 (SE = 0.006), *z* = −2.86, and a corresponding odds ratio of 0.983. This indicates that for each unit increase in HRV, the odds of making an error decrease by approximately 1.66%. These findings suggest that participants with better autonomic regulation, as indicated by higher HRV, tended to make fewer errors in the task. No other main effect of interactions reached significance.

### 3.3 Psychological Posture Effects II: RTs

RTs were analyzed using a linear mixed‐effects model with context (repeated, non‐repeated), posture (sitting, standing), difficulty (easy, difficult), and epoch (1–4) as fixed factors, leveraging trial‐wise data to fully capture across‐trial variability (see Baayen et al. 2008; Barr 2013; Judd et al. 2012). Once again, HRV, total PA, and BMI were included as covariates to account for individual differences in autonomic regulation, habitual PA levels, and body composition, respectively. Participant ID was included as a random factor, while context and posture were included as within‐subject factors with random slopes. A Type III analysis of variance with Satterthwaite's method was conducted on the full model.

We found a main effect of context, *F*(1, 35.2) = 28.66, *p* < 0.001, with faster responses to repeated (*M* = 1711 ms) compared to non‐repeated contexts (*M* = 1821 ms), demonstrating a robust CC effect of approximately 110 ms. There was no significant main effect of posture, *F*(1, 35.8) = 2.25, *p* = 0.142 (standing = 1725 ms; sitting = 1807 ms). Further, RTs were faster in the easy condition (*M* = 1383 ms) compared to the difficult condition (*M* = 2155 ms); the main effect of difficulty, *F*(1, 33.9) = 47.34, *p* < 0.001. The effect of the epoch factor was also significant, *F*(3, 17,452.6) = 20.47, *p* < 0.001, showing a decrease in RTs with increased time on task (epoch 1: 1834 ms; epoch 4: 1703 ms).

Of theoretical interest, we found a significant context × posture interaction, *F*(1, 169,69.3) = 12.27, *p* < 0.001, in addition to a significant three‐way context × posture × difficulty interaction, *F*(1, 169,69.5) = 6.90, *p* = 0.009. Decomposing the three‐way interaction by the difficulty factor by means of two separate analyses showed that the (two‐way) interaction between context and posture was non‐significant in the easy condition, *F*(1, 19.27) = 0.44, *p* = 0.514, but significant in the difficult condition, *F*(1, 8205.6) = 11.11, *p* < 0.001. Concerning the latter (difficult) search condition: While CC was significant during standing, *F*(1, 15.89) = 15.83, *p* = 0.001 (Non‐repeated: *M* = 2211 ms, Repeated: *M* = 2035 ms, CC effect = 176 ms), it was non‐significant with sitting position, *F*(1, 15.93) = 0.47, *p* = 0.503 (Non‐repeated: *M* = 2203 ms, Repeated: *M* = 2171 ms, CC effect = 32 ms).

### 3.4 Drift Diffusion Modeling

**FIGURE 2:**
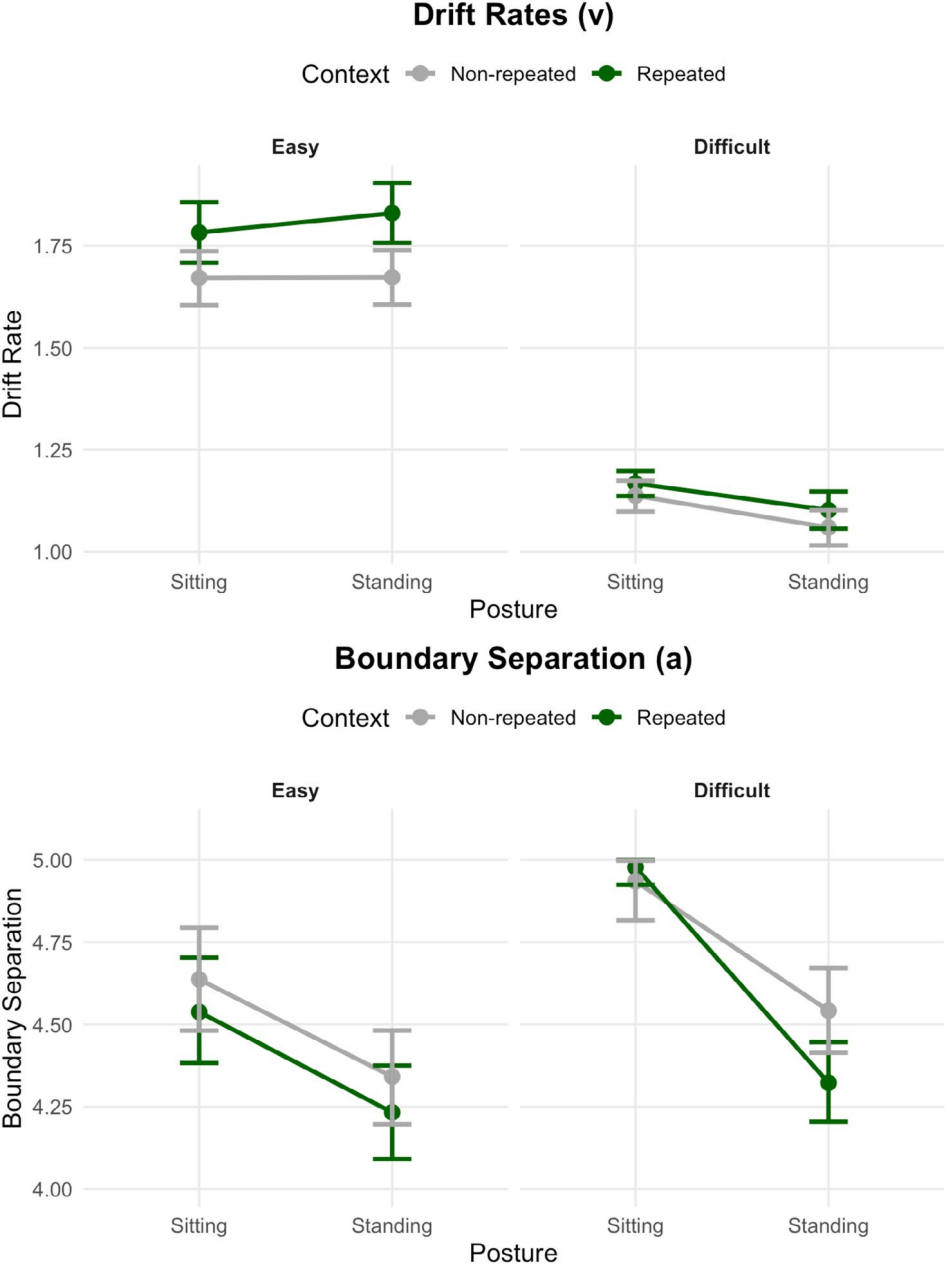
Drift rate (*v*) and boundary separation (*a*) parameter estimates of evidence accumulation models using the Dynamic Models of Choice (DMC) software (Heathcote et al. 2019), separately for the posture (sitting, standing), difficulty (easy, difficult), and context (repeated, non‐repeated) conditions. The error bars represent the 95% credible intervals, providing a measure of uncertainty around the parameter estimates.

Drift diffusion modeling revealed distinct patterns of effects on the speed of evidence accumulation (drift rate) and on decision thresholds (boundary separation; see Figure 2). For the *drift rates*, we observed three findings. First, there was a robust main effect of task difficulty (*M* = 0.604, 95% CI [0.565, 0.643], ER = Inf), with enhanced evidence accumulation in easy compared to difficult conditions. Second, there was a significant main effect of context (*M* = −0.087, CI [−0.124, −0.050], ER = 0), with increased drift rates in old‐context compared to new‐context trials. The context main effect was further qualified by a difficulty × context interaction (*M* = −0.097, CI [−0.169, −0.024], ER = 0.002) suggesting that drift‐rate differences between old‐context and new‐context displays were reliable with easy searches (standing: *M* = −0.160, CI [−0.249, −0.071], ER = 2.146e‐05; sitting: *M* = −0.110, CI [−0.201, −0.020], ER = 0.004), but not difficult searches (standing: *M* = −0.044, CI [−0.097, 0.010], ER = 0.028; sitting: *M* = −0.033, CI [−0.083, 0.015], ER = 0.049). Third, the main effect of posture was significant (*M* = −0.043, 95% CI [−0.080, −0.006], ER = 0.002), while the posture × context interaction was not significant (*M* = −0.031, 95% CI [−0.104, 0.043], ER = 0.196). However, there was a significant difficulty × posture interaction (*M* = 0.137, CI [0.064, 0.209], ER = 30,294.05). Follow‐up tests revealed that there was no strong evidence for a (sitting vs. standing) posture effect in the easy condition for repeated contexts (*M* = 0.051, 95% CI [−0.043, 0.145], ER = 7.817) or new contexts (*M* = 0.0004, 95% CI [−0.085, 0.084], ER = 1.030). However, there was strong evidence for a posture effect in the difficult condition for both repeated contexts (*M* = −0.105, 95% CI [−0.156, −0.055], ER = 2.971e‐05) and new contexts (*M* = −0.117, 95% CI [−0.168, −0.063], ER = 0), indicating expedited evidence accumulation with sitting compared to standing conditions. The three‐way interaction of difficulty × posture × context was not significant (*M* = −0.039, 95% CI [−0.184, 0.104], ER = 0.400).

Concerning the *boundary separation* parameter, we find the main effects of posture (*M* = −0.413, 95% CI [−0.499, −0.326], ER = 0, indicating lower thresholds when performing the search task with standing compared to sitting posture), difficulty (*M* = −0.259, 95% CI [−0.345, −0.171], ER = 0; due to lower thresholds in easy compared to difficult search conditions), and context (*M* = 0.090, 95% CI [0.003, 0.176], ER = 79.137; due to reduced thresholds in repeated compared to non‐repeated contexts). The three‐way interaction approached significance (*M* = −0.252, 95% CI [−0.585, 0.078], ER = 0.066). Separate analyses for the two difficulty conditions revealed that for the easy condition, the context × posture interaction was not significant (*M* = 0.009, 95% CI [−0.269, 0.278], ER = 1.140), and the effect of context approached significance (*M* = 0.101, 95% CI [−0.038, 0.240], ER = 15.201). This was different from the difficult condition, in which there was a significant context × posture interaction (*M* = 0.232, 95% CI [0.060, 0.403], ER = 346.694). Follow‐up tests showed that in this difficult condition, a CC effect in boundary separation was significant during standing (*M* = 0.216, 95% CI [0.061, 0.371], ER = 511.817; lower thresholds for repeated compared to non‐repeated contexts) but not during sitting (*M* = −0.058, 95% CI [−0.194, 0.043], ER = 0.140).

### 3.5 Recognition Test

Explicit memory for old‐context displays was examined by comparing participants' hit rates (old‐context display correctly judged as “old”) with their false‐alarm rates (new‐context display incorrectly judged as “old”) by means of a 2 (task difficulty: easy vs. difficult; between‐subject variable) × 2 (posture: sitting vs. standing; within‐subject variable) × 2 (response type: hits vs. false alarms; within‐subject variable) mixed‐design ANOVA. None of the main effects or interactions reached significance (all *p*'s > 0.15). The mean hit rate was 53.28%, compared to a false alarm rate of 49.53%. These findings suggest that participants could not reliably differentiate between repeated and non‐repeated displays.

## 4 Discussion

We tested whether perceptual difficulty and body posture influence CC of visual search, a central and well‐established effect in cognitive neuroscience. We had two questions: (1) Can we replicate previous (own) findings that showed that CC becomes weaker and takes longer to be established in the presence of a perceptually difficult T versus L letter search task? (2) How is CC affected when applying a posture manipulation that has been shown to influence executive functions and/or WM?

We approached these questions with a design in which we used the same type of letter stimuli in easy and difficult searches. In easy displays, participants searched for the target letter T among Ls. In difficult displays, non‐target Ls were presented with an offset, so they became more similar to the target letter T. Further, in both conditions, we examined old‐new RT differences in standing and sitting conditions, including physiological measurements. Moreover, DDM was applied to decompose the cognitive processes underlying contextual facilitation of aggregated search RTs on a more fine‐grained level while also allowing us to conduct a more direct comparison of the respective processes that may vary due to our posture and difficulty manipulations. We found that CC got weaker with difficult searches, but fully recovered when participants performed these searches with a standing posture. Cognitive modeling revealed that CC with difficult searches arises because the decision threshold was set more liberally, thus expediting response selection.

### 4.1 Effects of Target‐Distractor Similarity on CC

The first key finding extends and clarifies previous work, for example, by Darby et al. (2014), on the relationship between target‐distractor similarity and contextual learning. Darby and colleagues used T/L letters versus perceptually complex/detailed crayons to induce perceptual load as visual search items. By subtly offsetting line junctions in the L distractors while keeping all other stimulus parameters constant, we could replicate their findings while removing potential confounds, such as varying stimulus identities, when manipulating search task difficulty. Moreover, and going beyond previous CC studies that manipulated search difficulty (e.g., Darby et al. 2014; Vaidya et al. 2007; Lie 2023; Zinchenko et al. 2022), we found that, while repeated contexts enabled a higher drift rate (indexing faster evidence accumulation) in the easy condition, this “old‐context advantage” did not reliably emerge in the difficult condition. Here, participants struggled to exploit the repeated configuration to boost evidence accumulation when faced with more demanding target‐distractor discrimination. Returning to the observation that previous work has differed in mapping the drift rate to different theoretical CC process stages—target location or response selection (e.g., Chen et al. 2021; Sewell et al. 2018)—our findings support the former interpretation. Given that target appearance (an oriented T) and response requirements were identical across difficulty conditions, the observed difference in evidence accumulation rate (drift rate) likely stems from increased difficulty in target identification when distractors more closely resemble the target.

One plausible explanation for the overall weakened CC effect in difficult arrangements is that more complex distractors increase the perceptual load (e.g., Lavie 1995; Ahissar and Hochstein 1997), and thus substantial attentional resources need to be focused on the target item, which, in turn, may “overshadow” the ability to exploit the repeated spatial arrangements. This idea is supported by the findings from DDM that showed that across both—old and new –contexts, the difficulty manipulation (a) impaired the speed of evidence accumulation (decreased drift rate parameter) and (b) increased the decision thresholds during visual search, indexing more “cautious” responses.

### 4.2 Effects of Body Posture on CC

However, the notion of increased perceptual load and associated attention focusing on relevant—target—information falls short in explaining the results of our posture manipulation. Recall that the standing posture generally expedited visual search and improved accuracy across contexts and difficulty conditions, extending previous research investigating a link between postural states and visual search in general (e.g., Caron et al. 2023; Smith et al. 2019). More importantly, standing posture also led to context‐based facilitation of search RTs in difficult displays with high target‐distractor similarity. At first glance, this result is inconsistent with the above‐mentioned load studies, which assume that the processing of task‐irrelevant distractor (layout) information is determined fully by perceptual difficulty. Accordingly, finding a CC effect (which is inherently created through repeated distractor arrangements) during a perceptually difficult search performed with an upright standing posture would require extensions of load theory (e.g., Lavie 1995), such that CC, perceptual difficulty, and postural regulation interact.

An alternative might be to consider the standing posture as a secondary task. For instance, it has been shown that standing posture requires WM (e.g., Woollacott and Shumway‐Cook 2002). If so, performing the visual search task with a secondary (standing) task would increase *WM load* (e.g., for dual‐task scheduling). According to this view, and in contrast to the *perceptual* load account, participants would be less able to ignore irrelevant distractor items—see, for example, Lavie et al.'s (2004) load theory of cognitive control, which assumes that WM load and perceptual load lead to opposite effects on distractor processing. Accordingly, a CC effect may arise because irrelevant distractors are processed to a higher degree in standing compared to sitting posture. As a result, fewer items need to be searched in old‐context displays until the target item is selected and/or the repeated distractor context allows for faster target responses once it has been found. Critically, our model‐based analysis of mean RTs using DDM revealed that decreased decision thresholds, not DDM drift rates, produced the CC effect in difficult searches during standing posture. Further, drift rates were considerably lower in difficult searches (compared to easy searches), and even more importantly, they were statistically indistinguishable between old‐ and new‐context displays. Therefore, one must assume that the perceptual accumulation of target evidence (indexed by the drift rates) in old‐context displays is uninfluenced by the additional load coming from the posture manipulation. However, load‐induced processing of non‐target elements influenced response thresholds (indexed by the boundary separation), supporting a response‐based account of the CC effect (e.g., Kunar et al. 2007).

### 4.3 The Role of WM and Executive Processing

This specific account of cognitive load theory is difficult to distinguish from an account according to which body posture, rather than being a limiting factor in WM, may actually facilitate WM processing. The available evidence suggests that WM is a requirement for the brief maintenance of distractor‐target associations from LTM for the execution of (more effective) visual search in old‐context displays (e.g., Travis et al. 2013; Verschooren et al. 2021). Moreover, it has been shown (including the present study) that CC, measured under “default” sitting conditions, is reduced with difficult searches, probably because search demands dictate WM executive function requirements (e.g., Luria and Vogel 2011), which may weaken the WM‐based gating of sensory information and LTM contextual representations. However, it was also demonstrated that posture manipulations can up‐modulate WM executive processing (e.g., Busch et al. 2025; Dodwell et al. 2021), for example, by allowing more effective L‐T memory retrieval (of contextual representations). Here, we find evidence for this view: expectations in the form of acquired target‐distractor contextual LT memories can aid visual processing even when the search is difficult by allowing more effective, that is, liberal, responses. Arguably, our posture manipulation is likely to produce a facilitatory WM effect during response selection, assuming that WM can be divided into executive processes relating to the access of sensory representations held in WM and executive processes relating to the preparedness to act upon those sensory representations (e.g., Dodwell et al. 2021). Specifically, Dodwell et al. (2021) investigated how body posture (and moderate aerobic exercise) affect visual WM using a retro‐cue task (e.g., Griffin and Nobre 2003) combined with EEG measurements that index access and preparedness to WM information (i.e., contralateral delay activity, CDA, and stimulus‐locked lateralized readiness potentials, sLRP, components, respectively; cf. Töllner et al. 2013; see also Dodwell 2021). The study found that upright posture facilitated preparedness to act on WM information as indexed by the sLRP component. Applied to the present visual search task, it is thus possible that the standing posture improved participants' abilities to translate a stimulus into a response as specified in the task set. Assuming the Additive Factors Method as a valid method to decompose human information processing into a series of sequentially ordered processing stages (e.g., Sternberg 1969), then constraining the context factor by the posture factor is likely evidence that these factors operate on the same response‐selection stage of information.

### 4.4 Physiological and Psychological Arousal

It is worth noting that in our 2 (posture) × 2 (difficulty) × 2 (context) design, CC remained effective even after controlling for participants' BMI, PA level, and posture‐induced changes in HRV. This suggests that CC involves mechanisms that go beyond those captured by our physiological measures, such as arousal‐based mechanisms (e.g., Busch et al. 2025), and may instead reflect posture‐induced changes in sustained attentional states. In brief, it has been argued that upright standing may be associated with a more active engagement in the environment, potentially activating cognitive processes such as alertness and attentional selectivity (Smith et al. 2019)—aligning with our finding of reduced cautiousness (lowered response thresholds). It may be that such posture‐induced states are particularly effective in spatial arrangements that are subconsciously familiar through increased attentional selectivity when the search is demanding. Attentional states may also operate more directly through central neurometabolic and molecular processes that underlie response preparation and/or WM functions (Dodwell et al. 2021). However, the precise neurophysiological pathways through which posture exerts this influence remain to be fully elucidated, warranting further studies employing measures such as the stimulus‐locked lateralized readiness potential (sLRP) or contralateral delay activity (CDA) to map standing‐induced benefits onto specific neural substrates (Dodwell et al. 2021).

### 4.5 Drift Diffusion Model as an Account of Difficult Visual Search

In the present study, we modeled decision making in repeated versus novel T versus L letter search arrays that require eye movements (e.g., Zinchenko et al. 2025) using the drift diffusion model. Although increasingly applied in the more complex visual search literature (e.g., Sewell et al. 2018; Chen et al. 2021), these models were originally developed for elementary decisions and assume a single continuous accumulation process (e.g., Ratcliff and McKoon 2008). Applying them to visual searches that include multiple fixational decisions may entail interpretational caveats (Heathcote et al. 2019). This disadvantage is also reflected by the fact that the literature varies considerably in how the drift rate is mapped to theoretical CC process stages, that is, whether it represents target location or response selection (e.g., Sewell et al. 2018; Chen et al. 2021). Although the correct mapping remains an ongoing discussion, our results may be carefully taken as first evidence to validate DDM parameter interpretation for CC in visual search. What distinguishes the present study from previous modeling work on CC (e.g., Sewell et al. 2018) is its fully factorial design, encompassing four experimental conditions in which we systematically manipulated both perceptual load (difficulty) and postural load (standing vs. sitting) across old and new context arrays. We observed that DDM drift rates were overall lower in the difficult (vs. easy) T versus L letter search. Because the target‐response properties (i.e., the identity and response requirements for the target T) were held constant, and only the distractor properties varied across difficulty conditions, we interpret the drift rate as most strongly—though perhaps not exclusively—reflecting target search‐related processes in CC RTs.

Accordingly, for researchers applying a “simple” DDM to visual search tasks—assuming a single evidence accumulation process—our findings may be taken as support for the view that DDM drift rates primarily reflect search‐related perceptual processes, while boundary separation may capture response‐related processes in difficult visual search tasks (Kunar et al. 2007). Nevertheless, it remains crucial for future research to further validate these interpretations and to test them against alternative, as‐yet‐to‐be‐formalized models of CC in visual search, which posit not a single but multiple evidence accumulation processes—separately modeling accumulation for target versus non‐target decisions during the search phase and for orientation responses (left vs. right) during the response phase (see Wolfe 2021; Schwarz and Miller 2016). Importantly, such model comparisons require tailored experimental designs that enable tracking of both target and non‐target decisions, along with their corresponding RTs, during the search phase—an endeavor not possible with the data available for the present study.

## 5 Conclusion

Our results demonstrate that perceptual difficulty reduces contextual facilitation of search RTs, though additional postural states can bypass the detrimental effects of the high perceptual load on statistical contextual learning. Decomposing this effect through cognitive modeling revealed that CC in perceptually demanding search displays encountered in standing posture is due to lowered decision thresholds, thus supporting a response‐threshold account of the CC effect (e.g., Kunar et al. 2007). This result aligns with previous posture studies, which showed that standing speeds up response decisions in WM tasks. Assuming that CC also CC requires WM, the synergy between body posture and contextual repetitions is likely to happen at an executive response selection stage. When participants prepare to act upon learned contextual target templates held in WM, this synergy can yield performance improvements in old context displays even when a search is performed in a perceptually challenging environment.

## Author Contributions

**Artyom Zinchenko:** conceptualization, investigation, writing – original draft, validation, funding acquisition, methodology, visualization, writing – review and editing, formal analysis, project administration, data curation, supervision. **Nuno Busch:** writing – original draft, writing – review and editing, formal analysis, data curation. **Gordon Dodwell:** writing – review and editing, validation, supervision. **Thomas Geyer:** supervision, resources, project administration, writing – review and editing, validation, conceptualization, investigation, funding acquisition.

## Conflicts of Interest

The authors declare no conflicts of interest.

## Supporting information


Data S1.

## Data Availability

Experimental Material, Supporting Information, data, and analysis scripts will be made available on OSF upon acceptance of the manuscript: https://osf.io/evp28/

## Endnotes

^1^
In both experiments (1a and 1b), participants also performed two short cognitive control tasks (reported in Busch et al. 2025). The order of tasks was the same for each participant across the two (standing/sitting) sessions, but was counterbalanced between participants.
