# Supplementary material for: Withstand Context: Standing Posture Improves Contextual Cueing in Challenging Visual Search
Source: Psychophysiology. 2025 Jul 17;62(7):e70108. doi: 10.1111/psyp.70108 (PMC12272028; doi:10.1111/psyp.70108)
Supplement: Supplementary file 1 — Data S1. [file PSYP-62-e70108-s001.docx]

## Supplement

**Figure S1.** Trace plots for key parameters of the Drift Diffusion Model (DDM) fitted to the data. Rows represent different parameters: boundary separation (a), drift rate (v), starting point (z), and variability parameters (sz, sv, t0). Columns represent conditions defined by factors of difficulty (EASY vs. DIFFICULT), posture (SIT vs. STAND), and context (REP vs. NEW). Each trace shows posterior samples across iterations for individual chains, illustrating model convergence.


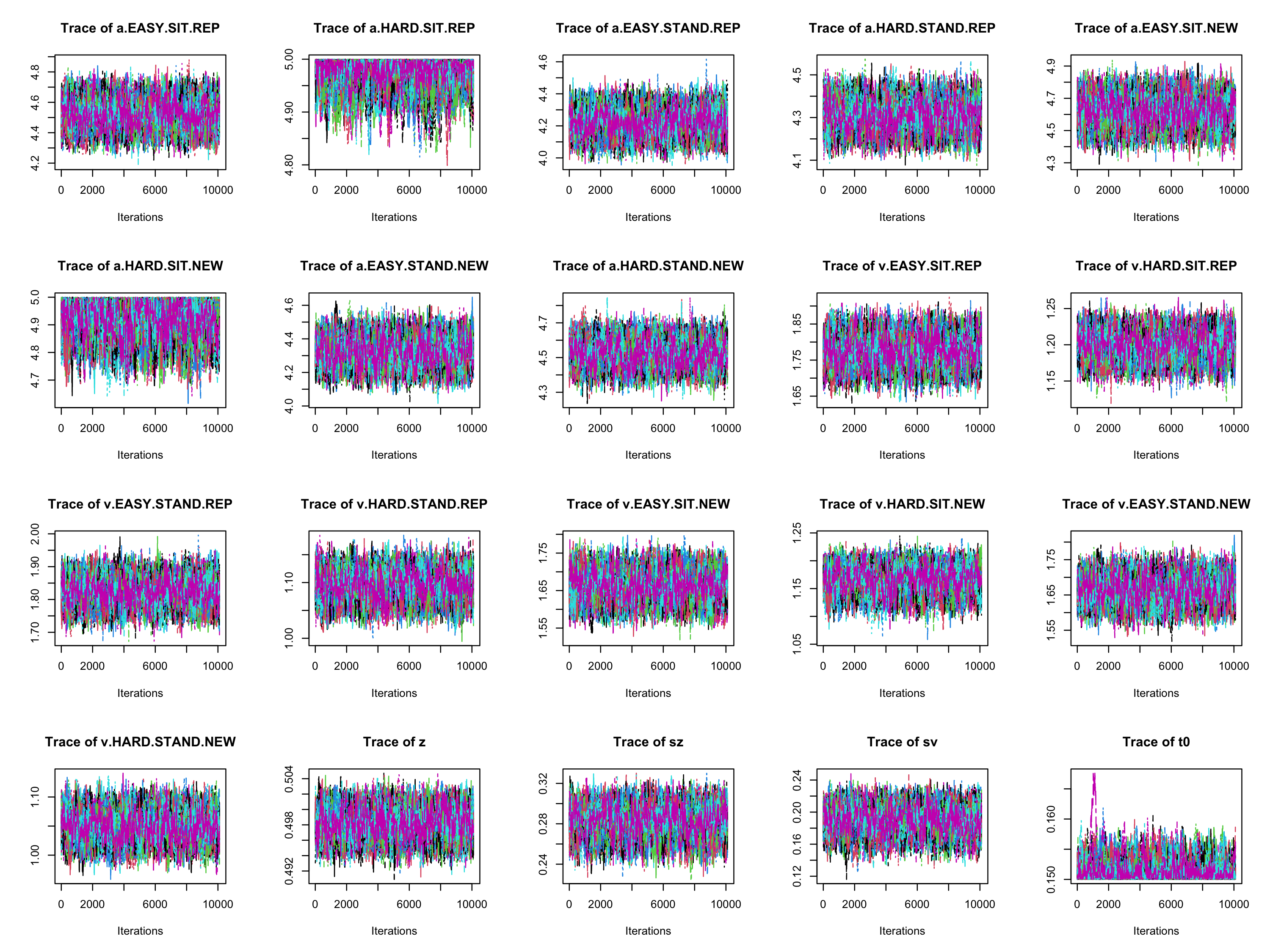


**Figure S2.** Cumulative distribution function (CDF) plot showing model fit for response times across all conditions. Lines represent model predictions (solid = left responses, dashed = right responses), while points represent observed data (circles = left responses, triangles = right responses). The precise alignment between model predictions and observed data indicates an excellent model fit. The x-axis shows response times in seconds, and the y-axis shows cumulative probability.


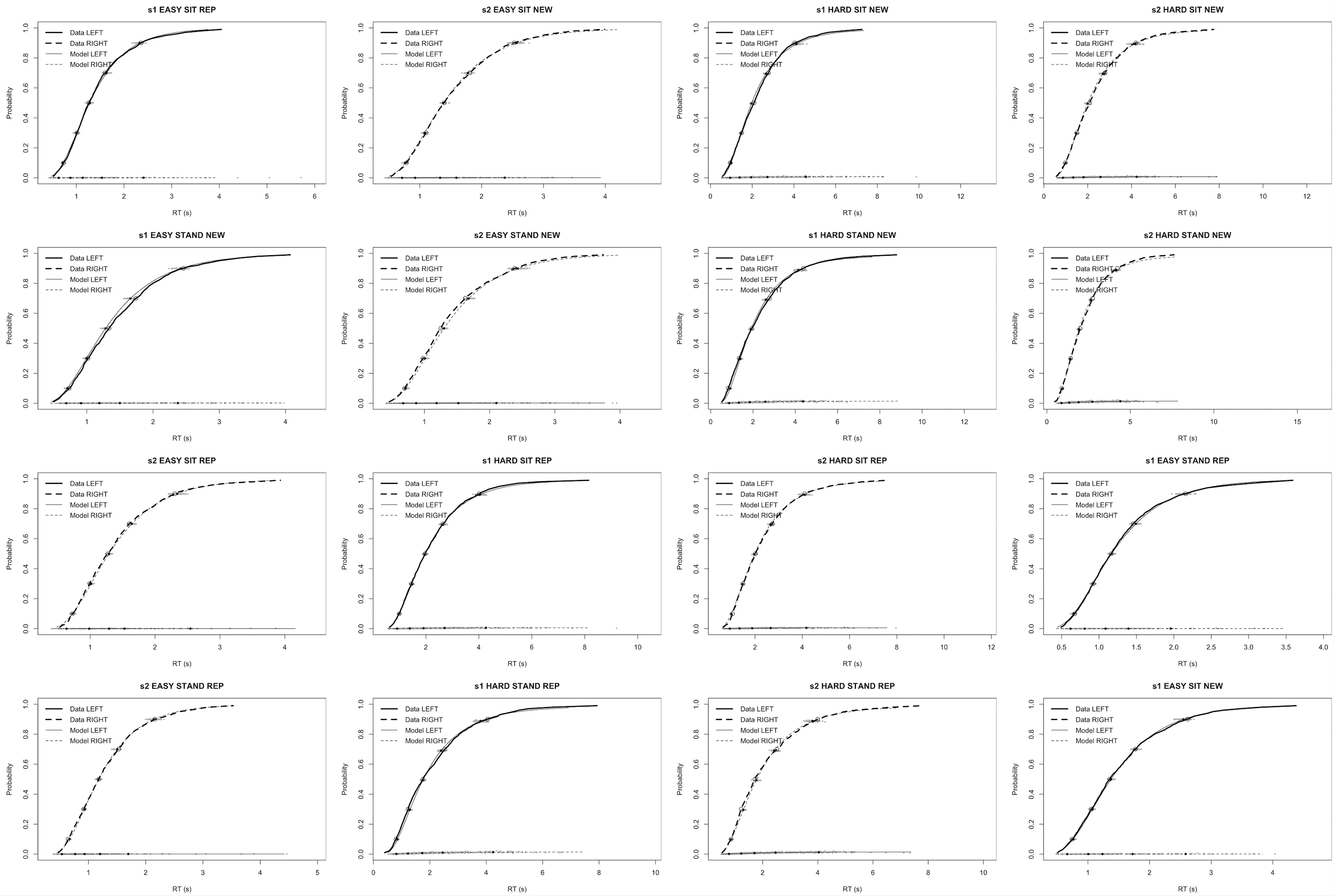


#### **S3: Diffusion-Decision Model (DDM) Selection and Validation.** To identify the most appropriate DDM for our Context × Posture × Difficulty design, we systematically compared multiple candidate models that differed in the number and combination of free parameters—drift rate (v), boundary separation (a), and non-decision time (t₀)—across the eight experimental conditions (cf. Chen et al., 2021). Based on our a priori hypotheses targeting both perceptual (v) and response-threshold (a) mechanisms, we focused our formal comparison on models with at least two free parameters. Thus, we evaluated three dual-parameter models (a + v, a + t₀, v + t₀) and one three-parameter model (a + v + t₀), each allowing their respective parameters to vary fully factorially across experimental cells. Model fitting and comparison followed the Dynamic Models of Choice (DMC) workflow (Heathcote et al., 2019). Model fit was primarily assessed using the Deviance Information Criterion (DIC), with lower DIC indicating a better trade-off between fit and complexity; for completeness, we also report AIC and BIC. We found that the best models for describing the RT data were the v + a and v + t₀ models, through estimates of t_0_ in the latter model collapsed to the lower bound of the prior distribution—first at 150 ms, then 100 ms, and finally at 0 ms as we relaxed prior constraints. This suggests that the v + t_0_ model accounted for fast responses by minimizing t₀ to implausibly low values. In such cases, parameter estimates cease to reflect psychologically meaningful processes and instead become boundary-induced artifacts of the fitting routine (e.g., Ratcliff & Tuerlinckx, 2002). Therefore, we considered only the theoretically plausible v + a model as an account of our RT data (for similar approaches, see, e.g., Heathcote et al., 2019; Chen et al., 2021).

#### Results of the 2, and 3-parameter models

| *Model* | DIC (×10⁶) | AIC (×10⁶) | BIC (×10⁶) |
| --- | --- | --- | --- |
| *v + t₀* | 3.076 | 3.073 | 3.073 |
| ***v + a*** | **3.078** | **3.073** | **3.073** |
| *a + t₀* | 3.273 | 3.118 | 3.118 |
| *v + a + t₀* | 4.154 | 4.144 | 4.144 |
